# Exploration of Antiproliferative Activity and Apoptosis Induction of New Nickel(II) Complexes Encompassing Carbazole Ligands

**DOI:** 10.1021/acsomega.3c01252

**Published:** 2023-03-22

**Authors:** Ramya Prabaharan, Ramesh Rengan, Sathiya Kamatchi Thangavel, Jan Grzegorz Małecki

**Affiliations:** † Centre for Organometallic Chemistry, School of Chemistry, Bharathidasan University, Tiruchirappalli 620 024, India; ‡ Department of Crystallography, Institute of Chemistry, University of Silesia, Katowice 40-006, Poland

## Abstract

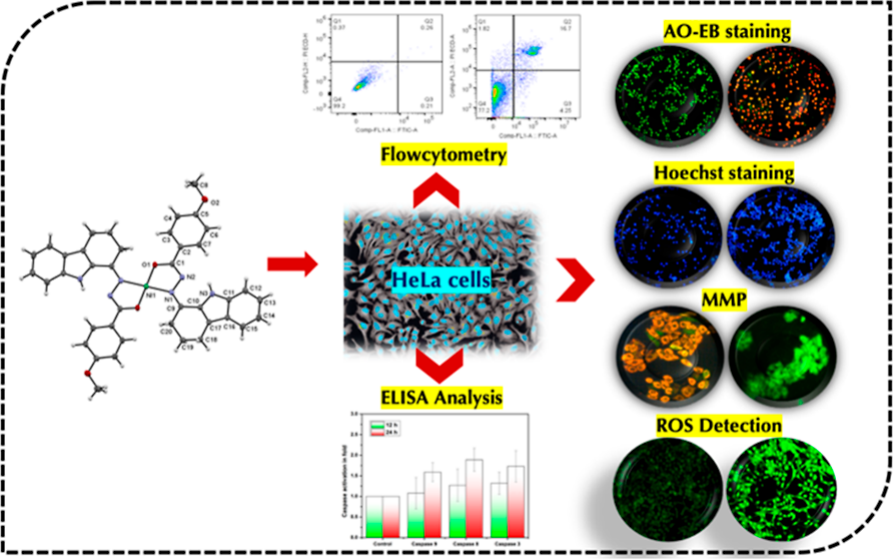

To attest the effectiveness of nickel complexes as anticancer drug candidates with minimum side effects, the present investigation describes the facile synthesis and anticancer activities of nickel(II) complexes enriched with three derivatives of carbazolone-based benzhydrazone ligands(L) having a [Ni(L)_2_] composition. Analytical and spectral techniques were used to characterize the synthesized Ni(II) complexes. The single-crystal X-ray diffraction performed for complex **4** confirmed the square planar geometry with a [Ni(κ^2^-N,O-L)_2_] arrangement. The MTT assay was carried out for the complexes to determine in vitro cytotoxicity against cancerous human-cervical carcinoma, human-colon carcinoma, and non-cancerous L929 (fibroblast) cells. All three complexes exhibited good toxicity against the cancer cells with a low IC_50_ concentration. Complex **4**, containing −OCH_3_ fragment, exhibits high lipophilicity and revealed exceptional cytotoxicity against cancer cells. AO-EB fluorescent staining indicated apoptosis-associated cell morphological changes after exposure to complex **4**. The apoptosis induction was further confirmed by a HOECHST-33342 fluorescent staining technique via chromosomal condensation and nuclear fragmentation. Further, reactive oxygen species (ROS) and mitochondrial membrane potential (MMP) mechanistic studies revealed that complex **4** can raise ROS levels and reduce MMP and promote mitochondrial dysfunction-mediated apoptotic cell death. Further, stimulation of late apoptosis by complex **4** in cervical cancer cells was quantitatively differentiated through the staining of phosphatidylserine externalization by flow cytometry. Furthermore, the ELISA analysis confirmed that complex **4** induced apoptosis through caspase activation.

## Introduction

Cisplatin and its descendants, oxaliplatin and carboplatin, administered to cancer patients, exhibit many side effects, such as osteoporosis, neurological problems, nephrotoxicity, liver diseases, and so forth.^1^ Hence, many researchers have been engrossed in finding an efficient transition-metal-based drug with various types of ligands that possess much lesser side effects than platinum drugs.^2−7^ In recent times, biologically active Ni(II) complexes have been synthesized and their cytotoxicity toward cancer cells was investigated.^8−10^ Further, in our body, nickel is a micronutrient that helps in protein synthesis and present in higher amounts in nucleic acids.^11^ It resides at the active sites of numerous classes of important enzymes like urease, hydrogenase, and CO dehydrogenase. Further, being a member of the platinum group, nickel may replicate features of platinum complexes, including coordination geometry, kinetics of substitution, oxidation states, and affinity toward different ligands. Hence, researchers rekindled their attention to nickel(II) coordination complexes for finding out their mode of action toward cancer cells.^12,13^ Hydrazones, the distinct class of Schiff base when combined with different groups of atoms, have numerous pharmaceutical applications.^14^

**Figure 1:**
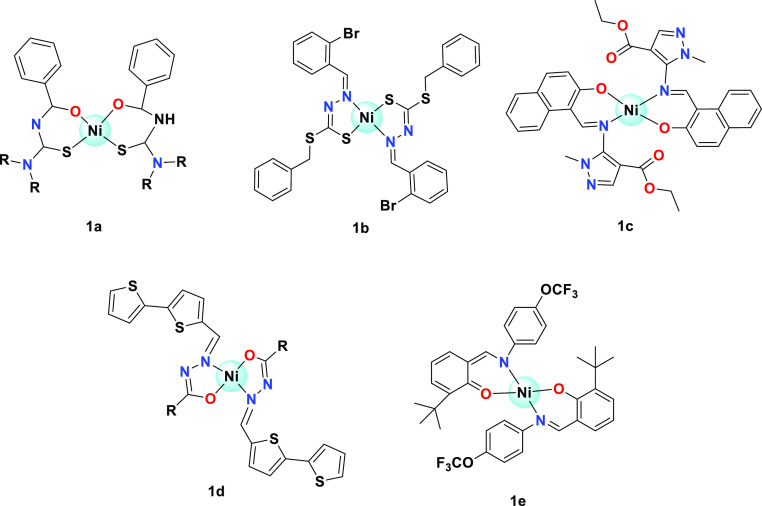
Previous reports on biological activity of Ni(II) square planar complexes.

Six Ni(II) square planar complexes with benzoyl thiourea derivatives (Figure 1a) have been reported and investigated for their cytotoxicity against the T47D cell line.^15^ Square planar Ni(II) complexes containing *S*-benzyldithiocarbazate (Figure 1b) and their in vitro cytotoxicity against MKN45 and HepG2 cancer cell lines were studied by Qiu and et al. They have reported the IC_50_ concentration about 2–43 μg mL^–1^.^16^ Ni(II) square planar complexes containing the pyrazolone carboxylate moiety (Figure 1c) were synthesized and examined their antiproliferative activity against HepG2, MGC80-3, T-24, BEL-7404, NCI–H460, SK-OV-3, A549, and HL-7702 cells. The reported IC_50_ concentrations were about >100 to 13.26 μM.^17^ Yang and his co-workers have explored the pharmaceutical properties of nickel(II) complexes (Figure 1d) comprise aroylhydrazone ligands. The in vitro cytotoxicity against MCF-7 and human-cervical carcinoma (HeLa) cells was studied and the IC_50_ concentrations were about 86–125 μM.^18^ In vitro biological evaluation of nickel(II) complexes containing 2-(((4-trifluoromethoxy) phenylimino)methyl)-6-*tert*-butylphenol (Figure 1e) have been documented by Rambabu et al.^19^

As an extension of our earlier research toward the exploration of anticancer activity of nickel complexes,^20,21^ herein, we report three new nickel(II) square planar complexes with carbazolone benzhydrazone ligands. The structures of all the synthesized nickel complexes have been recognized by analytical and spectral techniques. A single-crystal X-ray diffraction (XRD) method was used to confirm the molecular structure of the complex. The anticancer property of the newly synthesized complexes was studied by the in vitro cytotoxicity method against the cancerous cells, such as HeLa and human-colon carcinoma (HT-29). Additionally, different biochemical assays, like AO-EB, HOECHST 33342, reactive oxygen species (ROS), MMP, and flow cytometry, were performed to study the cell death mechanism.

## Results and Discussion

**Scheme 1:**
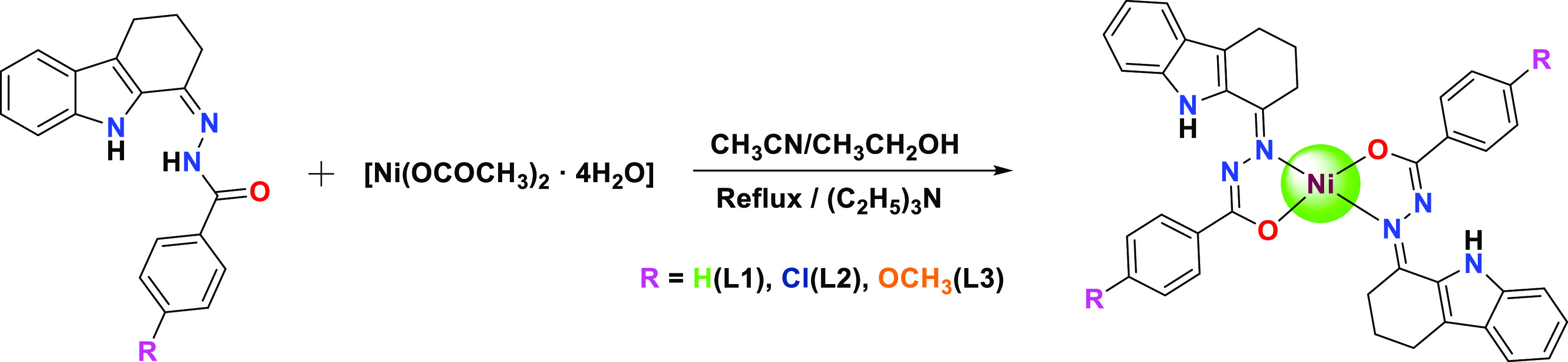
Synthesis of Ni(II) Carbazolone Benzhydrazone Complexes

The carbazole-based benzhydrazone ligands were prepared by following our previous reports. The reaction of 2,3,4,9-tetrahydro-1H-carbazol-1-one and derivatives of benzhydrazide in ethanol was carried out at room temperature for 3 h and a pale yellow residue was obtained. The green needle crystals of the new Ni(II) complexes were recovered by the reaction of Ni(CH_3_COO)_2_·4H_2_O with the prepared ligands in the ratio of 1:2, respectively, for 6 h in 1:1 ethanol and acetonitrile under reflux conditions. The addition of a few drops of triethylamine accelerates the reaction by enabling amido–imidol tautomerization, thus forming a five-member chelate ring through the bonding of azomethine nitrogen and imidolate oxygen with Ni ions. The obtained new Ni(II) complexes were with the general formula [Ni(L)_2_] (Scheme 1).

**Figure 2:**
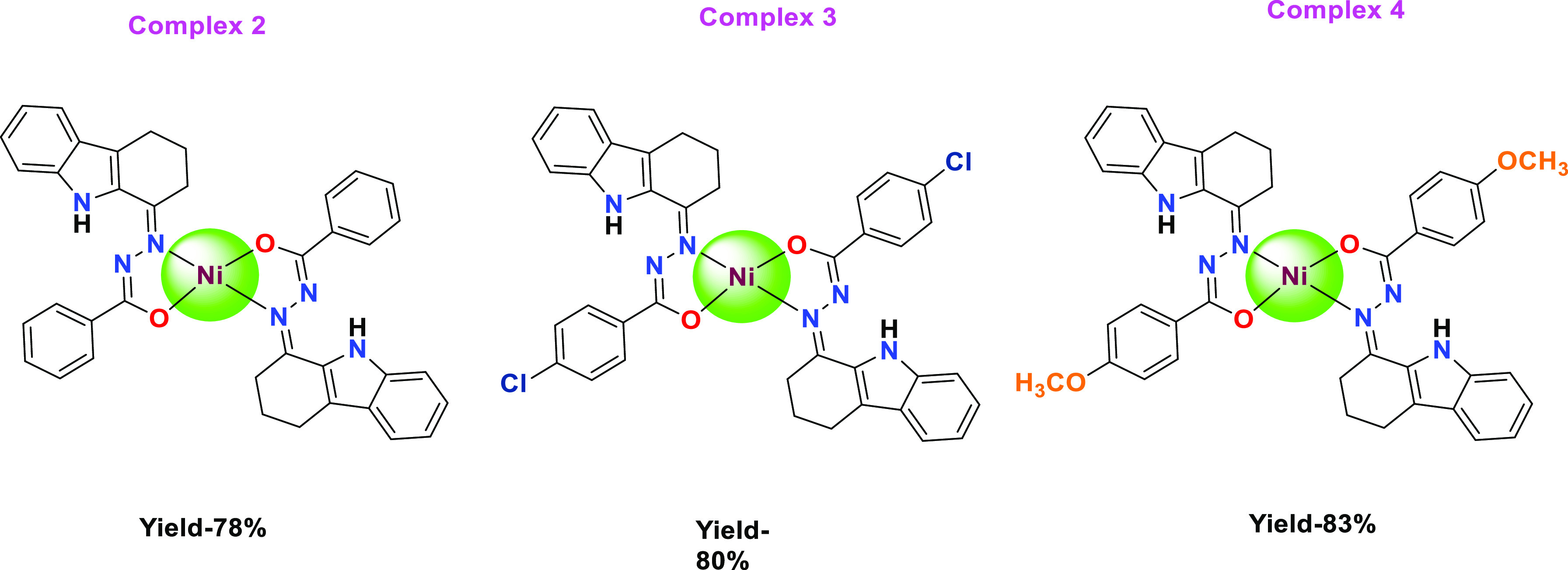
Newly synthesized Ni(II) square planar complexes.

The complexes were found to be air-stable, non-hygroscopic, and soluble in several organic solvents like CHCl_3_, CH_2_Cl_2_, CH_3_CN, DMF, DMSO, and so forth. FT-IR spectroscopy was used to confirm the functional groups present in the complexes. The free ligands exhibited two peaks at regions of 3458–3336 and 3234–3054 cm^–1^ that correspond to ν(N–H) functional groups. Another peak was observed at 1644–1680 cm^–1^, assignable to the ν(C=O) functional group. While for the complexes, the peaks at ν(C=O) and a ν(N–H) were disappeared, indicating the enolization. A new peak at 1363–1371 cm^–1^ corresponding to ν(C–O) was found for all the complexes that represent the coordination site via imidolate oxygen after deprotonation. Besides, substantial decrement in stretching frequency of ν(C=N) up to 75 cm^–1^ was found in the complexes. This validates another coordination of the ligand via azomethine nitrogen to the metal ion.^20^ From the above results, bidentate coordination of the ligand via imidolate oxygen and azomethine nitrogen to the metal Ni(II) ion was confirmed (Figure 2).

The UV–visible spectra of all the newly synthesized complexes (Figures S1–S3) were recorded at room temperature in an acetonitrile medium between 200–800 nm. The observed bands were highly intense around 240–340 nm for the ligands as well as the complexes. In addition, the MLCT transitions were emerged with medium intensity at 366–380 nm in spectra of the complexes. ^1^H NMR spectra of all the complexes were recorded in DMSO and are unsuccessful with very broad bands.^20^ However, mass spectra for all the complexes were obtained in an acetonitrile solvent (Figures S4–S6) and confirm the formation and compositions of the new Ni(II) carbazolone benzhydrazone complexes. All the synthesized Ni(II) complexes **2–4** display peaks corresponding to [M-L]^+^ fragments at *m*/*z* ratios of 382.0551, 394.1660, and 413.0650, respectively.

**Figure 3:**
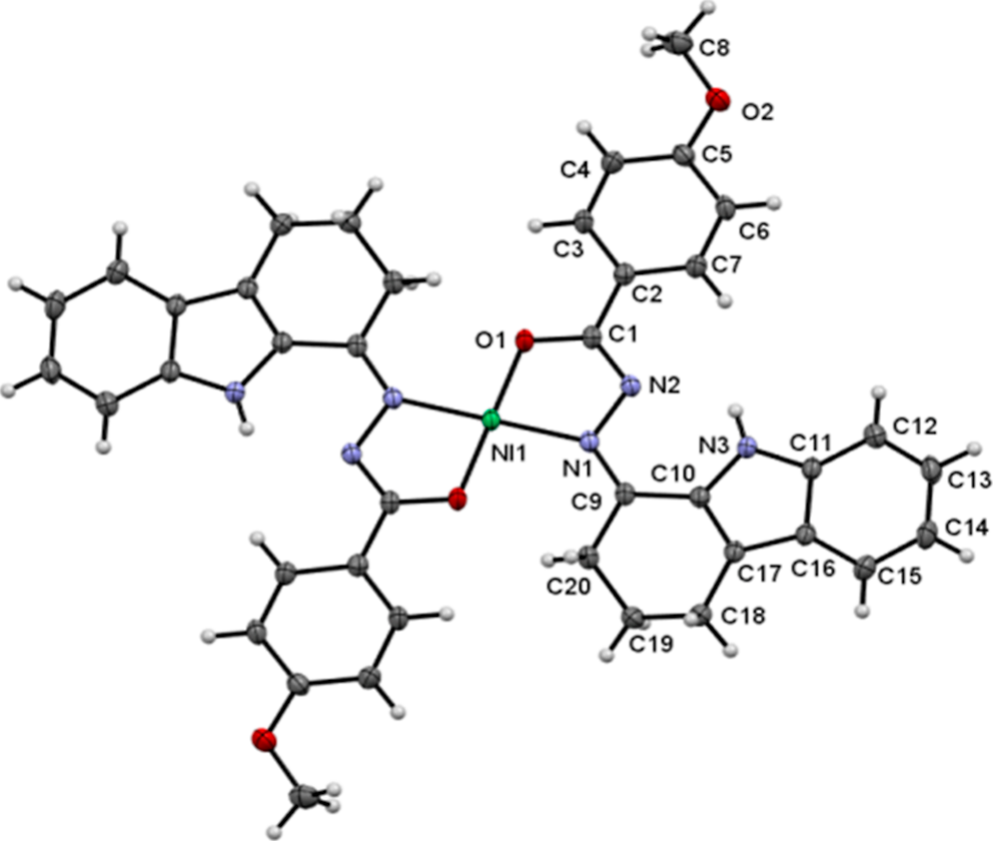
ORTEP diagram of complex **4** with 30% probability. All the hydrogen atoms were omitted for clarity. The bond angles (deg.) around the Ni(1) ion: O(1)–Ni(1)–N(1) = 83.00(6), O(1)–Ni(1)–O(1) = 180.0, and N(1)–Ni(1)–N(1) = 180.0. Bond lengths (Å): Ni(1)–N(1) = 1.9446(14) and Ni (1)–O(1) = 1.8264(12).

Further attempts were taken to grow single crystals for all the complexes by the slow evaporation method in the presence of dichloromethane/acetonitrile (2:1) solvent combination. Single crystals of suitable quality for one of the complexes (complex **4**) were obtained and the ORTEP view of the complex is displayed in Figure 3. The crystal data and refinement parameters are presented in Table S1 and the selected bond lengths and angles are displayed in Tables S2 and S3. The space group of the crystallized complex was found to be *P*2_1_/*c*. The central Ni(II) metal ion was surrounded and coordinated by two carbazolone benzhydrazone ligands through (N, O) azomethine nitrogen and imidolate oxygen and thus forms a square planar geometry. Two five-member chelate rings with the Ni(II) metal ion were generated by tetra-coordination of the ligands. The bite angles around Ni(II) ions are O(1)–Ni(1)–N(1) = 83.00(6)^o^, O(1)–Ni(1)–O(1) = 180.0 °, N(1)–Ni(1)–N(1) = 180.0 °. The Ni(1)–N(1) and Ni(1)–O(1) bond lengths surrounding the Ni(II) center were found to be 1.9446(14) and 1.8264(12) Å, respectively. The bond lengths and bond angles were in good accordance with other reported Ni(II) square planar complexes.^17−21^

### Stability Studies

Stability assessment is significant for pharmaceutical development of new compounds to measure the quality of the drugs. The efficiency and safety of the drugs are also essential for new drug development and clinical prosperity. Using UV–visible spectroscopy, the stability studies were performed for all the synthesized Ni(II) complexes in aqueous media. The media used in this assessment was dimethylsulfoxide (1%) in a phosphate buffer solution with a pH of 7.4 and all the complexes were prepared to 1 × 10^–3^ M concentration. Spectra were recorded for different time laps till 72 h, and the corresponding information is provided in Supporting Information—S12. The spectra did not display any noticeable change in either position or intensity of the bands over 72 h. Consequently, all the Ni(II) complexes were subjected to further biological examinations as all are stable.^20,21^

### Calculation of Partition Coefficient (log *P*)

The permeability of the drug molecules into a lipid cytomembrane is a prime initiative for designing drugs and its development. The main properties like absorption, transportation, and expulsion of the drugs take control over the chief pharmacokinetic properties. Accordingly, we resolve the permeability of all the complexes with the help of calculated log *P* values using a *n*-octanol–water system. Based on the solubility of the complexes in aqueous and organic phases, the log *P* values are determined.^22−24^ The calculated log *P* values for the complexes (**2–4**) are 0.86 ± 0.14, 1.57 ± 0.12, and 1.95 ± 0.05, respectively (Table 1). The obtained log *P* values evidently uncovers that all the complexes showed good lipophilicity. Even though the complexes exhibit the same structural properties, the log *P* values are not the same which is due to the presence of different substituents on the ligands. Complexes **3** and **4** display higher permeability characters (log *P* value) due to the presence of halogen and electron-donating group, respectively. Additionally, the presence of the carbazole moiety in the complexes enhanced the lipophilicity.^25,26^

**Table 1:** In Vitro Cell Viability of the Carbazole Ligands, Ni(II) Carbazolone Benzhydrazone Complexes, and Cisplatin over 24 and 48 h Incubation Periods; IC_50_(μM) ± Standard Deviation and log *P* (Lipophilic Character)

	IC_50_ (μM)					
	24 h			48 h		
compounds	HeLa	HT-29	L929	HeLa	HT-29	L929
L1	>100	>100	>100	>100	>100	>100
L2	>100	>100	>100	86.23 ± 1.12	94.03 ± 1.27	>100
L3	>100	>100	>100	73.41 ± 0.82	81.74 ± 1.09	>100
**complex 2**	49.27 ± 1.05	68.59 ± 0.27	248.14 ± 1.12	40.12 ± 0.09	61.25 ± 0.47	236.31 ± 1.45
**complex 3**	45.44 ± 0.72	59.16 ± 0.64	253.08 ± 0.61	32.78 ± 1.19	50.80 ± 1.11	247.43 ± 0.28
**complex 4**	43.39 ± 0.21	57.42 ± 0.09	275.76 ± 0.35	28.15 ± 0.32	41.25 ± 1.28	269.05 ± 1.10
cisplatin	28.04 ± 0.18	34.28 ± 1.45	146.51 ± 0.79	12.36 ± 1.81	14.27 ± 1.57	141.21 ± 0.37

### Determination of Cell Viability and Cytotoxicity

To study the ability of the prepared ligands and the synthesized complexes in retarding the growth of cancer cell, the MTT assay was performed with human cervical carcinoma (HeLa), human colon carcinoma (HT-29), and non-cancerous L929 (fibroblast) cell lines. As a reference, cisplatin is also examined with all the cell lines for an incubation period of 24 h and as stated in Table 1. The ligands and the nickel precursors did not show any significant inhibition to the growth of the cancer cells till 100 μM concentrations. Hence, this proves that the chelation of the ligands with metal ions plays a vital role in cytotoxicity.

Table 1 reveals that the complexes exhibit a good cytotoxic effect against HeLa and HT-29 cancer cells. The IC_50_ concentration of complex **4** was found to be lower than the other two complexes. This is due to the electron-donating group (−OCH_3_) present in complex **4** that influences the lipophilic nature of the nickel complex. This helps its permeability through the cell membranes as demonstrated by its greater log *P* value. Between HeLa and HT-29 cell lines, the antiproliferative effect of HeLa cells by the complexes was found to have a greater extent than HT-29 cells. Hence, IC_50_ values confirm that the synthesized complexes are specific toward cancerous cells.^20,27^ It has been observed that all the complexes exhibit an increase of cytotoxicity against the cell lines when the impact of contact period of the complexes on the proliferations of cell lines in 48 h. Hence, the nickel complexes demonstrated time-dependent cytotoxicity on the viability of cancerous cells. Henceforth, further investigations were carried out with complex **4** to examine the mechanism of cancer cell death using HeLa cells.

### Cell Death Mechanism by Dual AO/EB (Acridine Orange-Ethidium Bromide) Staining

**Figure 4:**
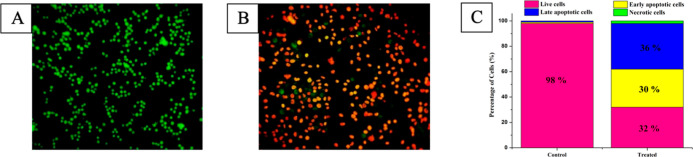
AO-EB dual staining of HeLa cancer cells. (A) Control; (B) cells administered with complex **4** (IC_50_ concentration) for 24 h; and (C) percentage of live and apoptotic cells.

The AO/EB double staining method via fluorescence-activated cell sorting (FACS) can be envisioned to detect apoptosis-associated morphological changes in cellular membranes.^28^ This method distinguishes live cells, early, late apoptotic, and necrotic cells using fluorescence properties and cell morphological characteristics.^29^ HeLa cancer cells were stained with acridine orange and ethidium bromide. Usually, EB stains impaired cellular membranes whereas AO stains both alive and impaired cells. Live and active cells emit green fluorescence, but necrotic cells emit total red-orange fluorescence evenly. On the contrary, due to DNA fragmentation and chromosomal condensation, the early apoptotic cells fluoresce with orange patches. The control portion not administered with test complex **4** emits bright green evenly (Figure 4A). After administering the IC_50_ concentration of complex **4** into HeLa cells along with AO-EB dual stains, EB passes into the cell and displays an orange tint by overwhelming AO stain (Figure 4B). As seen in Figure 4B, complex **4** stimulates significant apoptosis in the cancer cells.^30^

### HOECHST 33342 Staining to Detect Apoptosis

**Figure 5:**
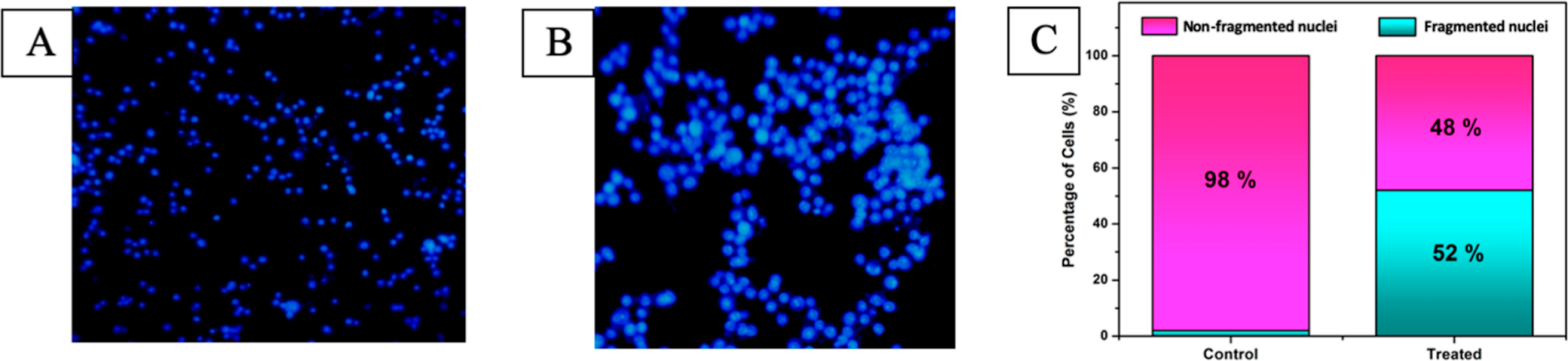
HOECHST staining of HeLa cancer cells. (A) Control; (B) cells administered with complex 4 (IC_50_ concentration) for 24 h; and (C) percentage of nonfragmented and fragmented cells.

Hoechst 33342 staining is another technique that reveals the morphological changes of the cancer cells before and after treating with the test complex. Control cells (Figure 5A) and the cells treated with an IC_50_ concentration of complex **4** were stained with Hoechst 33342. Bright blue fluorescence patches were observed for the complex treated cells after 24 h (Figure 5B). This is due to the structural changes (chromosomal condensation and nuclear fragmentation) that happened attributed to apoptosis.^31^

#### ROS Detection

**Figure 6:**
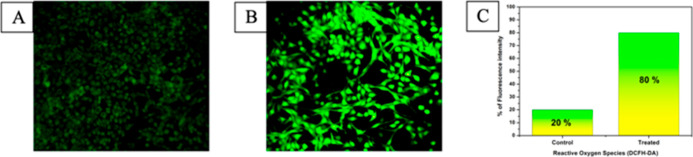
ROS detection of HeLa cancer cells. (A) Control; (B) cells administered with complex **4** (IC_50_ concentration) for 24 h; and (C) fluorescent intensities based on ROS generation.

ROS, like hydrogen peroxide, superoxide anion, and hydroxyl radical, are produced on mitochondrial oxidative metabolism along with cell’s reaction toward cytokines and bacterial invasion. At low levels of ROS, it could participate in cell signaling. Whereas, at higher levels they might damage (DNA and RNA) and engage in apoptosis via mitochondrial dysfunction.^32^ The quantity of ROS is detected with the help of oxidant fluorescent dye. The nonpolar dye DCFH-DA turned out to be deacetylated and transformed into DCFH (polar) by intracellular esterases. However, DCFH is not identified using a microscope. Hence, they require ROS to change them into DCF, which highly emits a fluorescence.^33^ No fluorescence was observed in the control (Figure 6A), whereas complex **4** administered HeLa cells fluoresce bright green (Figure 6B). This indicates complex **4** triggers apoptosis (programmed cell death) in cancer cells via ROS generation.

#### Detecting Mitochondrial Membrane Potential

**Figure 7:**
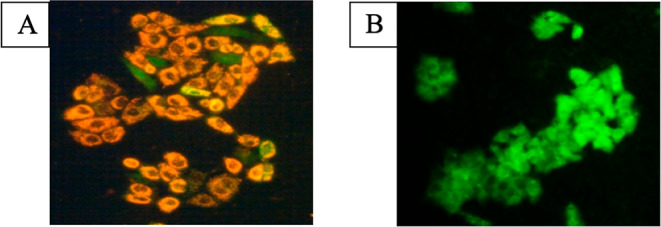
JC-1 staining of HeLa cancer cells. (A) Control and (B) cells administered with complex **4** (IC_50_ concentration) for 24 h.

The mitochondrial dysfunction-mediated apoptosis was assayed via ROS generation. After the mitochondrial impairment, the mitochondrial membrane potential (MMP) is reduced and the same was examined using JC-1 fluorescence dye. The dye is used to assay the MMP to determine mitochondrial viability. Proapoptotic factors and other apoptotic inducing features were produced by mitochondria, which serves to be a significant role for apoptosis. When JC-1 dye is stained on the cells, it reveals membrane potential-dependent accretion in mitochondria via the changes in fluorescence from red orange (∼590 nm) to green patches (∼525 nm).^34^ In the control group (Figure 7A), JC-1 accumulates and produces a red fluorescence correlating to a high MMP. Figure 7B shows that after 24 h of incubation of HeLa cells with complex **4**, JC-1 exhibits a green fluorescence. The variation in fluorescence color displays the decrease in MMP, indicating apoptosis on administering the nickel(II) complex.

#### Flow Cytometry

**Figure 8:**
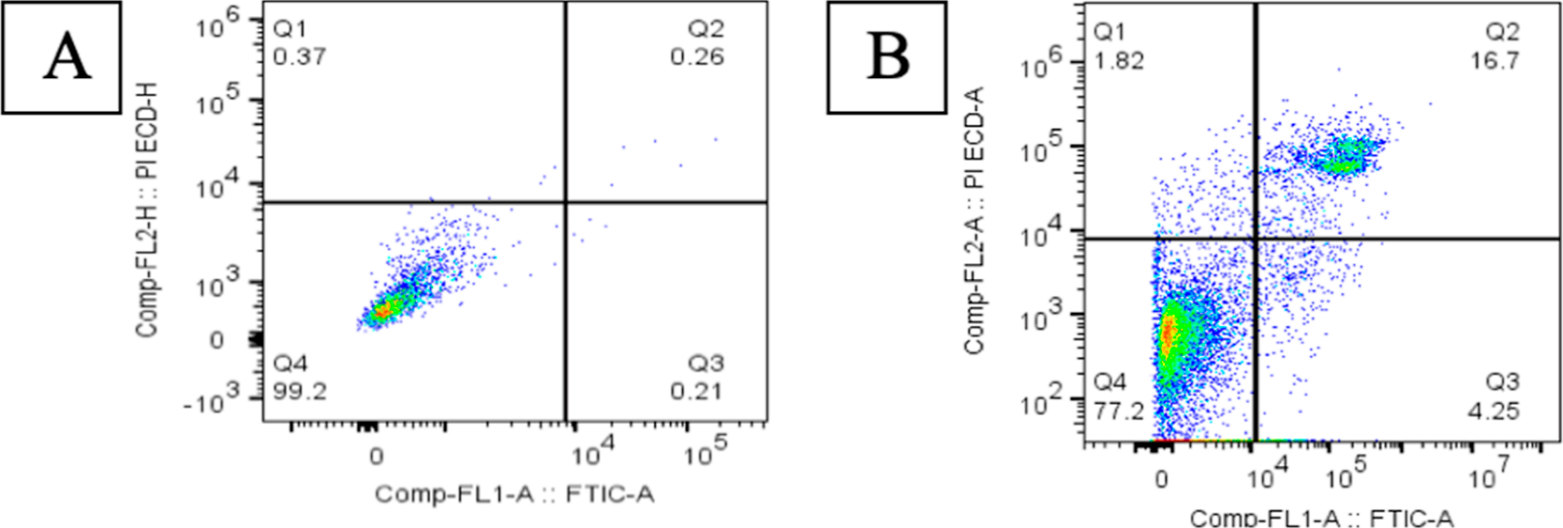
Double staining method of HeLa cancer cells (A) control and (B) cells administered with complex **4** (IC_50_ concentration) for 24 h.

Our previous studies on the cell death mechanism demonstrated that the new Ni(II) complexes significantly triggered apoptosis in HeLa cells through a mitochondrial dysfunction pathway. A precise quantification of apoptosis, on the other hand, is important to better understand the function and effectiveness of potential anti-cancer drug candidates. Apoptosis can be assessed quantitatively via the double staining method using annexin V-FITC and PI (propidium iodide). The phospholipid—PS (phosphatidylserine) is normally found in the inner layer of the cell membrane of healthy cells. Whereas, its exposure toward the outer side happens at the time of apoptosis. Hence, the PS location is useful for determining the morphological changes of the cell by its binding with annexin V.^35^ Additionally, propidium iodide is stained to distinguish and quantify the live cells from dead cells. First, quadrant Q4 (lower left) manifests live cells via annexin V(−)/PI(−) staining. Second, quadrant Q1 and third, quadrant Q2 (lower right and upper right) expresses early and late apoptotic cells using annexin V(+)/PI(−) and annexin V(+)/PI(+) staining, respectively. In each quadrant, the division of apoptotic cells were shown (Figure 8A,B). When HeLa cells were administered with complex **4** by its IC_50_ concentration for 24 h, different apoptotic features were observed. Figure 8B demonstrates that when HeLa cells are subjected to nickel complex **4**, the proportion of early apoptotic cells is only about 1.82%, whereas the proportion of late apoptotic cells is approximately 16.7%. The cell population in the Q2 quadrant [annexin V(+)/PI(+)] was higher (16.7%) than the control (Figure 8A), demonstrating the induction of late apoptosis.^36^

### Assessment of Caspase-3/8/9 Activation

**Figure 9:**
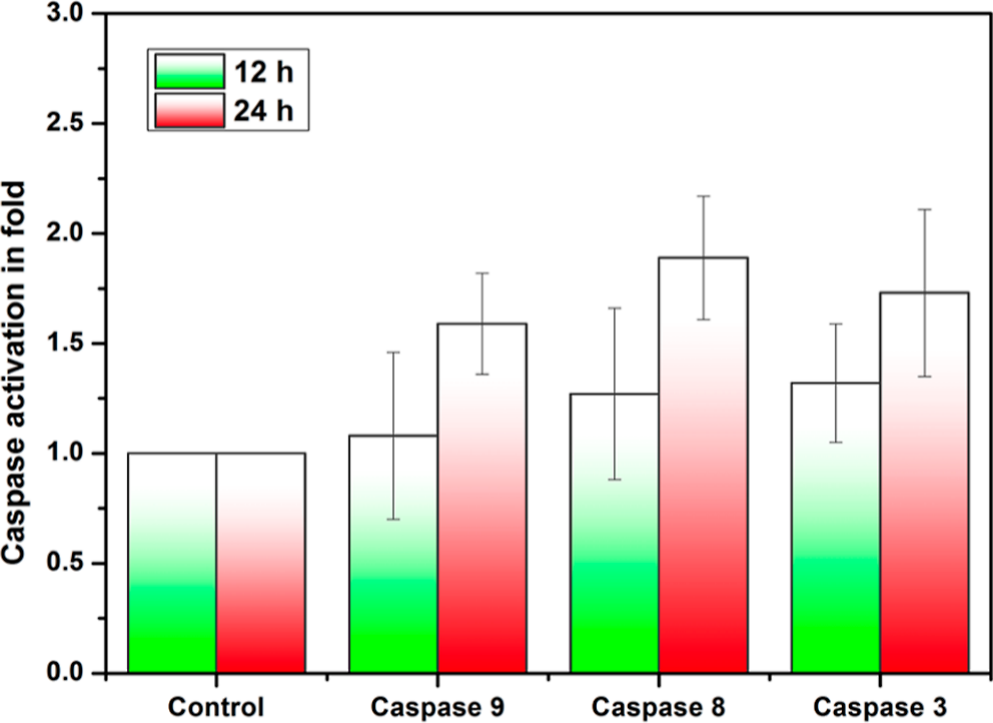
Effect of complex **4** on caspase—3, 8, 9 in HeLa cells by the ELISA assay for 12 and 24 h.

The apoptotic mode of cancer cell death was further substantiated by caspase activation by ELISA analysis. In eukaryotes, caspases(protease enzymes) play a significant role in induction, transduction, and amplification of intracellular apoptotic signals. Based on the amino acid composition, caspases are divided into three sub-families, namely, activator, executioner, and mediator caspases.^37^ Most importantly, the initiator and executioner caspases get involved in the apoptotic signaling cascade. Caspase-8 and -9 are the initiators of the extrinsic and intrinsic pathways of apoptosis, respectively. Both mechanisms converge in the activation of the executioner caspase-3.^38^ Hence, the IC_50_ concentration of complex **4** at different time intervals (12 and 24 h) were treated on HeLa cells with caspases-3, 8 and 9. The outcomes confirmed that complex **4** efficiently upregulated the expression of caspase-8 among other caspases 3 and 9 for triggering cancer cell apoptosis. The results also showed a time-dependent increase in caspase activity than control cells (Figure 9).^39^

## Conclusions

The scarcity of metal-based drugs for treating cancer, the second prime cause of death globally has led to new anticancer metallodrugs. However, there are only few reports available in nonprecious nickel-based benzhydrazone complexes as anticancer agents. Hence, this research focuses on synthesizing three new nickel(II) complexes incorporating bioactive carbazolone benzhydrazone ligands that possess good anticancer activity. The synthesized complexes exhibit a [Ni(κ^2^-N,O-L)_2_] arrangement elucidated by elemental, various spectral analyses, and single-crystal XRD. Different biochemical assays were performed to investigate in vitro anticancer activity of the new nickel complexes. Further, all the complexes displayed enhanced cytotoxicity against the cervical and colon cancer cells screened than the free carbazolone benzhydrazone ligands. In particular, complex **4** exhibits potent activity against cervical cancer cells with lower IC_50_ values (43.39 ± 0.21 μM) without affecting the non-cancerous L929 cells (275.76 ± 0.35 μM). The outcomes of the AO-EB and HOECHST 33342 fluorescent staining assays unveil that the cytotoxicity of the nickel complex was triggered by apoptosis in cancerous cells. The increase in the ROS level and decrease in mitochondrial membrane potential by the nickel complex **4** witness that the programmed cell death was mediated by mitochondrial dysfunction. Flow cytometry analysis confirms the quantitative differentiation of late apoptosis by the translocation of phosphatidylserine. In addition, the apoptotic mode of cell death was confirmed by caspase-8 enzyme activation. The cancer target specificity and apoptosis induction behaviors of the present nickel complexes open up the prospect of promoting efficient nickel-based anticancer drug candidates over platinum and ruthenium drugs.

## Experimental Section

Materials, methods, and crystal data collection are given in the Supporting Information.

### Preparation of Carbazolone Benzhydrazone Ligands

The carbazole-based benzhydrazone ligands have been prepared in an ethanol medium by mixing 2,3,4,9-tetrahydro-1*H*-carbazol-1-one (1 mmol) and derivatives of benzhydrazide (1 mmol). The reaction was carried out in a few drops of concentrated hydrochloric acid at ambient temperature for 3 h. The pale yellow residue has been obtained and washed with diethyl ether.^25^

### Synthetization of Ni(II) Square Planar Complexes with Benzhydrazone Ligands

The new Ni(II) complexes were synthesized by reacting Ni(CH_3_COO)_2_.4H_2_O (0.05 mmol) and carbazolone benzhydraone ligands (0.1 mmol) in the ratio 1:2, respectively. The reactions were performed using a 1:1 ethanol/acetonitrile medium in the presence of a few drops of base Et_3_N under reflux conditions. The acquired green precipitates were crystallized by the slow vaporization technique using CH_2_Cl_2_/acetonitrile combination. The obtained green crystals were washed with petroleum ether and dried in vacuum.

### Characterization of Complexes (**2–4**)

#### [Ni(L1)_2_] (**2**)

Green. Yield = 78%; decomposition temperature—325 °C; Calcd C_38_H_32_N_6_NiO_2_: C, 68.80; H, 4.86; N, 12.67%. Found: C, 68.71; H, 4.85; N, 12.64%.IR (KBr, cm^–1^): 2944 ν(N–H), 1522 ν(C=N), 1368 ν(C–O). UV–vis (CH_2_Cl_2_): λ_max_, nm (ε_max_ dm^3^ mol^–1^ cm^–1^): 224 (6568), 276 (1466), 366 (1787). ESI-HRMS: calcd for: *m*/*z* 360.0647 [M – L]^+^; found, *m*/*z* 382.0551 [M – L – H + Na]^+^.

#### [Ni(L2)_2_] (**3**)

Green. Yield = 79%; decomposition temperature—334 °C; Calcd C_38_H_30_Cl_2_N_6_NiO_2_: C, 62.33; H, 4.13; N, 11.48%. Found: C, 62.24; H, 4.12; N, 11.44%. IR (KBr, cm^–1^): 2930 ν(N–H), 1523 ν(C=N), 1363 ν(C–O). UV–vis (CH_2_Cl_2_): λ_max_, nm (ε_max_ dm^3^ mol^–1^ cm^–1^): 234 (7438), 293 (3178), 380 (3421). ESI-HRMS calcud for: *m*/*z* 394.0257 [M – L]^+^; found: *m*/*z* 394.1660 [M – L]^+^.

#### [Ni(L3)_2_] (**4**)

Green. Yield = 83%; decomposition temperature - 329 °C; Calcd C_40_H_36_N_6_NiO_4_: C, 66.41; H, 5.02; N, 11.62%. Found: C, 66.34; H, 5.01; N, 11.59%. IR (KBr, cm^–1^): 2930 ν(N–H), 1507 ν(C=N), 1371 ν(C–O). UV–vis (CH_2_Cl_2_): λ_max_, nm (ε_max_ dm^3^ mol^–1^ cm^–1^): 241 (1830), 284 (1012), 375 (2063). ESI-HRMS: calcd for: *m*/*z* 390.0752 [M – L]^+^; found: *m*/*z* 413.0650 [M – L + Na]^+^.

## Supporting Information Available

Complete details of materials, methods, crystal data collection crystallographic data, refinement parameters, selected bond lengths, bond angles, UV–vis spectra, HRMS spectra, and stability studies of the complexes (PDF)
CCDC numbers for complex **4** is **2113259** (CIF)

## Supplementary Material

ao3c01252_si_001.pdf

ao3c01252_si_002.cif
