# Supplementary material for: Exploration of Antiproliferative Activity and Apoptosis Induction of New Nickel(II) Complexes Encompassing Carbazole Ligands
Source: ACS Omega. 2023 Mar 22;8(13):12584–91. doi: 10.1021/acsomega.3c01252 (PMC10077545; doi:10.1021/acsomega.3c01252)
Supplement: Supplementary file 1 — ao3c01252_si_001.pdf [file ao3c01252_si_001.pdf]

Supporting Information

Exploration of Antiproliferative Activity and Apoptosis Induction of New Nickel(II) Complexes Encompassing Carbazole Ligands

Ramya Prabaharan,[a] Sathiya Kamatchi Thangavel,[a] Ramesh Rengan,*[a] and Jan Grzegorz Małecki[b]

[a]Centre for Organometallic Chemistry, School of Chemistry, Bharathidasan University, Tiruchirappalli – 620 024, India.

[E-mail: ramesh_bdu@yahoo.com and phone No. 0431- 2407053;
Fax: 0091-431-2407045]

[b]Department of Crystallography, Institute of Chemistry, University of Silesia, 40-006, Katowice, Poland.

CONTENTS

1 Materials, Methods and Crystal data collection..S2

2 Experimental Procedures.. S3-S5

3 Table of crystal data, selected bond lengths & angles for complex **4**……. S6-S8

4 UV-vis spectra of the complexes **2 - 4** S9

5 HR-MS spectra of complexes **2 - 4**. S10-S12

6 Stability studies of the complexes **2 - 4**. S13

7 References...S14

1. Materials, Methods and Crystal data collection

Best commercial grade reactants and solvents were used for all the reactions. Nickel(II) acetate tetrahydrate and benzhydrazide derivatives were purchased from Merck and Aldrich Chemicals and used as received. 2,3,4,9-tetrahydro-1H-carbazol-1-one was prepared from the procedures as specified in the literature.[1] Boeties micro heating table was used to record the melting points and are uncorrected. The analysis of carbon, hydrogen, nitrogen and sulphur were performed at Sophisticated Test and Instrumentation Centre (STIC), Cochin University of Science and Technology, Kochi. Perkin-Elmer 597 spectrophotometer was utilized to record the IR spectra of ligands and complexes with KBr pellets within the range of 4000-400 cm^{-1}. A Cary 300 Bio UV–vis Varian spectrophotometer was used to record the electronic spectra of complexes in the range 800-200 nm. A Micro mass thermo-scientific HRMS-Exactive Plus EMR instrument was utilized for High Resolution Mass Spectrometry of the complexes. Single crystal of complex **4** was grown by slow evaporation of a dichloromethane in acetonitrile solution at room temperature. A single crystal of suitable size was covered with Paratone oil, mounted on the top of a glass fibre, and transferred to a Bruker AXS Kappa APEX II single crystal X-ray diffractometer using monochromated MoK_{α} radiation (λ = 0.71073). Data were collected at 293 K. The structure was solved by direct methods using SIR-97 and was refined by the full matrix least-squares method on F2 with SHELXL-97.[2] Non-hydrogen atoms were refined with anisotropy thermal parameters. All hydrogen atoms were geometrically fixed and collected to refine using a riding model. Frame integration and data reduction were performed using the Bruker SAINT Plus (Version 7.06a) software. The multi-scan absorption corrections were applied to the data using SADABS software.[3] Figure 3 was drawn with ORTEP and the structural data have been deposited at the Cambridge Crystallographic Data Centre: CCDC **2113259**.

2. Experimental Procedures

Stability studies

UV-visible time dependent spectral method has been used to examine the stability of the complexes. Complexes were dissolved in a minimum amount of 1% DMSO and then diluted with PBS buffer to 1×10^{-3} M concentration. The hydrolysis profiles of the complexes were monitored by their electronic spectra over 72 h.

Partition coefficients determination

The "shake-flask" technique was used to evaluate the lipophilicity of complexes **2 - 4** using octanol/water phase partitions. Double distilled water and analytical grade octanol (Sigma Aldrich) were used to produce octanol-saturated water and water-saturated octanol phases. Complexes **2−4** (1 mg/mL; ethanol/water) have been diluted to 2, 4, 6, 8, and 10 μg/mL in water and alternatively were diluted to 2, 4, 6, 8, and 10 μg/mL in octanol, respectively. Equal volumes (50/50) of the complexes in the proper concentrations (4 mg/mL) have been shaken for 24 hours at ambient temperature. The organic and aqueous fractions have been separated and centrifuged when equilibrium was attained. Finally, UV-visible spectroscopy was used to determine the complex concentration in each phase. The concentration of the sample solution was used to calculate the partition coefficients (log P) by using the equation log P = log[(1−3)oct/(1−3)aq].[4]

Cell culture

The cell lines HeLa, HT-29, and L929 were collected from ATCC and the stock cells were cultured in DMEM/ F12 enriched with 10% inactivated Fetal Bovine Serum (FBS), penicillin (100 IU/ml), streptomycin (100 μg/ml) in a humidified atmosphere of 5% carbon dioxide at 37°C until confluent. The cells were dissociated with cell dissociating solution (0.2 % trypsin, 0.02 % EDTA, 0.05 % glucose in PBS). The viability of the cells have been checked and centrifuged. Further, 50,000 cells /well of Jurkat was seeded in a 96 well plate and incubated for 24 hours at 37°C, 5 % carbon dioxide incubator.

MTT assay

The monolayer cell culture has been trypsinized and the cell count was altered to 1.0 x 10^5 cells/mL using respective media comprising 10% FBS. 100 µL of the diluted cell suspension (50,000 cells/well) was added to each well of the 96 well microtiter plate. When a partial monolayer formed after 24 hours, the supernatant was swiped off and the monolayer has been washed with the medium. 100 µL of various concentrations of complexes have been added on to the partial monolayer in microtiter plates. Then, the plates have been incubated at 37 ^oC for 24 hours in 5% carbon dioxide atmosphere. After incubation, the test solutions in the wells have been discarded and 100 µL of MTT (5 mg/10 ml of MTT in PBS) was added to each well. The plates have been incubated for four hours at 37 ^oC in 5% carbon dioxide atmosphere. The supernatant has been removed and 100 µL of DMSO was applied to dissolve the formed formazan. The absorbance was recorded using a microplate reader at 590 nm. The % growth inhibition has been determined using the following formula

Cell viability (%) = (Absorbance of each well/ Absorbance of control well) × 100

AO-EB Staining method

AO-EB fluorescent double staining has been employed to examine the apoptosis in cancer cells after the incubation with the complex **4**. Briefly, cells were seeded in 24-well plates at a density of 4000 cells/well and incubated at 37 °C for 24 hours. Then the test complex **4** in their IC_{50} µM concentrations were incubated with HeLa cells. The staining solution (10 µL) comprising AO (100 µg/mL) and EB (100 µg/mL) was applied to each well (500 µL) after 24 hours. A fluorescence microscope (Olympus, CKX-53, Japan) was employed to visualize the cells. The % of viable and dead cells has been measured in at least 3 random fields of microscope.

Hoechst 33342 staining method

5 × 10^5 HeLa cells have been incubated with IC_{50} concentration of the complex **4** for 24 hours in a 6-well culture plate and have been fixed with 4% paraformaldehyde followed by permeabilization with 0.1% Triton X-100. Cells were then stained with 50 µg /mL Hoechst 33342 for 30 min at ambient temperature. The cells undergoing apoptosis were observed and visualized by epifluorescence microscope (Carl Zeiss, Germany).

Reactive Oxygen Species (ROS) Assay

To quantify the intracellular ROS, 5 × 10^5 HeLa cells have been seeded on a 6-well plate comprising cover slip and incubated overnight for attachment. After incubation, the cells have been treated using fresh medium with IC_{50} concentration of the complex **4** and incubated further for 24 hours. After that, the cover slip has been removed from the culture plate, and stained with 40 µM of 2',7'-dichlorofluorescein-diacetate (DCFHDA) dye for half an hour. The stained cover slip has been rinsed using PBS solution and imaged under fluorescence microscope.

Mitochondrial Membrane Potential (MMP)

Culture cells on cover slips in 6-well cell culture plates at a density around 5 x 10^5 cells/mL overnight in an incubator (5% carbon dioxide, 37 °C). Complex **4** (IC_{50} µM) has been incubated with HeLa cells for 24 hours. 1-10 µM JC-1 working stock in cell culture medium has been prepared. For 1µM working stock, 5 µL of JC-1 (200 µM) has been added per 1 mL cell culture medium. Then the plates have been incubated (5% carbon dioxide, 37°C) for 15-30 minutes. The cells were immediately examined under a fluorescent microscope (Olympus, CKX-53, Japan), and the proportion of dead cells was calculated in at least three randomly chosen microscopic fields.

Annexin V-FITC/PI staining by flow cytometry method

HeLa ells have been seeded in a 6-well plate (10^5 cells/well)) and cultured at 37 °C for 24 hours. The cells have been treated with complex **4** (IC_{50} concentration) and incubated for 24 hours. Then, the cells were trypsinized, washed with PBS and stained with annexin V-FITC/PI according to the annexin V-FITC apoptosis detection kit. Finally, apoptosis induction has been assessed using a flow cytometer (SYSMEX, Japan), and the data have been analyzed by Flow Jo software. The cells that were not treated were employed as a control group.

Caspase activity assay

The caspase activity assay was determined by a chromogenic assay using caspase-3 (Calbiochem, Merck), caspase-8 and 9 (Chemicon International Inc.) colorimeter activity assay kits. Briefly, after treatment of complex **4** at different time intervals (12 and 24 h), 1.5 106 cells were harvested, lysed with cell lysis buffer (50 mM HEPES, 100 mM NaCl, 0.1%

CHAPS, 1 mM DTT, 100 mM EDTA) followed by centrifugation at 10 000 rpm for 1 min. About 50 ml of supernatant was incubated with specific substrate (at 37°C) for 2 h in a water bath. The absorbance of the cleaved substrate was measured at 405 nm using a microtiter plate reader (BioRad, UK).

3. Table S1. Crystal data and refinement parameters for complex 4

Empirical formula	$C_{40}H_{36}N_6NiO_4$
Formula weight	723.46
Temperature/K	295(2)
Crystal system	monoclinic
Space group	$P2_1/c$
a/Å	9.5193(5)
b/Å	6.6063(3)
c/Å	26.6880(12)
α/°	90
β/°	90.800(4)
γ/°	90
Volume/Å^3	1678.17(14)
Z	2
ρ_{calc}g/cm^3	1.432
μ/mm^{-1}	0.632
F(000)	756.0
Crystal size/mm^3	0.36 × 0.16 × 0.06
Radiation	Mo Kα (λ = 0.71073)
2Θ range for data collection/°	6.882 to 58.72
Index ranges	-11 ≤ h ≤ 12, -8 ≤ k ≤ 6, -35 ≤ l ≤ 29
Reflections collected	11463
Independent reflections	4067 [R_{int} = 0.0290, R_{sigma} = 0.0341]
Data/restraints/parameters	4067/0/237
Goodness-of-fit on F^2	1.029
Final R indexes [I>=2σ (I)]	R_1 = 0.0371, wR_2 = 0.0834
Final R indexes [all data]	R_1 = 0.0536, wR_2 = 0.0917
Largest diff. peak/hole / e Å^{-3}	0.35/-0.33

Table S2. Bond lengths (Å) for the complex 4

Atom	Atom	Length/Å	Atom	Atom	Length/Å
Ni1	O1[1]	1.8264(12)	C4	C5	1.378(3)
Ni1	O1	1.8264(12)	C5	C6	1.383(3)
Ni1	N1[1]	1.9446(14)	C6	C7	1.373(3)
Ni1	N1	1.9446(14)	C9	C10	1.446(2)
O1	C1	1.2989(19)	C9	C20	1.513(2)
O2	C5	1.366(2)	C10	C17	1.385(2)
O2	C8	1.423(3)	C11	C12	1.397(2)
N1	N2	1.418(2)	C11	C16	1.411(3)
N1	C9	1.316(2)	C12	C13	1.368(3)
N2	C1	1.292(2)	C13	C14	1.393(3)
N3	C10	1.380(2)	C14	C15	1.381(3)
N3	C11	1.366(2)	C15	C16	1.402(3)
C1	C2	1.479(2)	C16	C17	1.424(2)
C2	C3	1.380(2)	C17	C18	1.490(3)
C2	C7	1.396(2)	C18	C19	1.513(2)
C3	C4	1.384(3)	C19	C20	1.524(3)

Table S3. Bond angles (°) for the complex 4

Atom	Atom	Atom	Angle/˚	Atom	Atom	Atom	Angle/˚
O1^{1}	Ni1	O1	180.0	C7	C6	C5	119.96(17)
O1	Ni1	N1	83.00(6)	C6	C7	C2	121.32(17)
O1	Ni1	N1^{1}	97.00(6)	N1	C9	C10	125.64(15)
O1^{1}	Ni1	N1	97.00(6)	N1	C9	C20	120.07(15)
O1^{1}	Ni1	N1^{1}	83.00(6)	C10	C9	C20	114.22(15)
N1	Ni1	N1^{1}	180.0	N3	C10	C9	127.77(16)
C1	O1	Ni1	112.85(11)	N3	C10	C17	108.68(15)
C5	O2	C8	117.96(16)	C17	C10	C9	123.40(16)
N2	N1	Ni1	110.56(10)	N3	C11	C12	130.59(18)
C9	N1	Ni1	135.59(12)	N3	C11	C16	107.69(15)
C9	N1	N2	113.78(13)	C12	C11	C16	121.72(17)
C1	N2	N1	110.13(14)	C13	C12	C11	117.35(19)
C11	N3	C10	109.52(16)	C12	C13	C14	122.01(18)
O1	C1	C2	117.30(15)	C15	C14	C13	121.25(19)
N2	C1	O1	123.07(16)	C14	C15	C16	118.23(19)
N2	C1	C2	119.62(15)	C11	C16	C17	107.14(15)
C3	C2	C1	120.28(16)	C15	C16	C11	119.44(17)
C3	C2	C7	117.69(16)	C15	C16	C17	133.39(18)
C7	C2	C1	122.02(16)	C10	C17	C16	106.94(15)
C2	C3	C4	121.52(17)	C10	C17	C18	123.83(15)
C5	C4	C3	119.73(18)	C16	C17	C18	129.22(16)
O2	C5	C4	124.77(17)	C17	C18	C19	108.63(15)
O2	C5	C6	115.42(16)	C18	C19	C20	112.99(15)
C4	C5	C6	119.78(17)	C9	C20	C19	114.80(15)

4. UV-vis spectra of the complexes 2 - 4

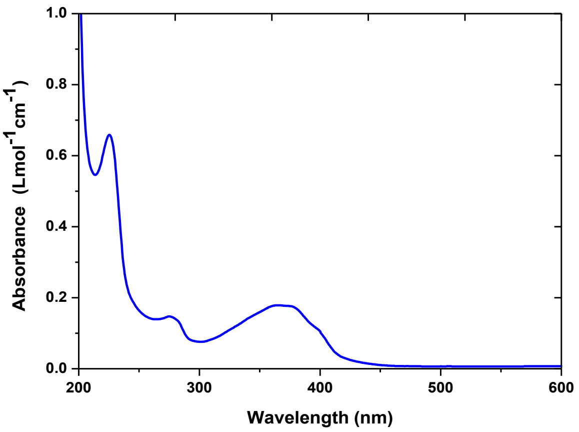

Figure S1. UV-vis spectrum of complex 2 [A_{max} (nm): 366, 276, 224]

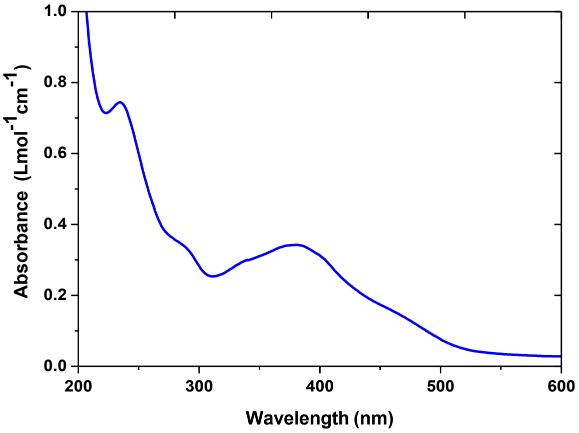

Figure S2. UV-vis spectrum of complex 3 [A_{max} (nm): 380, 293, 234]

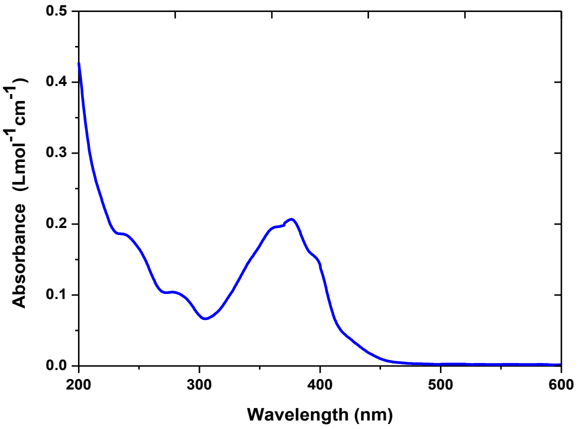

Figure S3. UV-vis spectrum of complex 4 [A_{max} (nm): 375, 284, 241]

5. HR-MS spectra of complexes 2-4

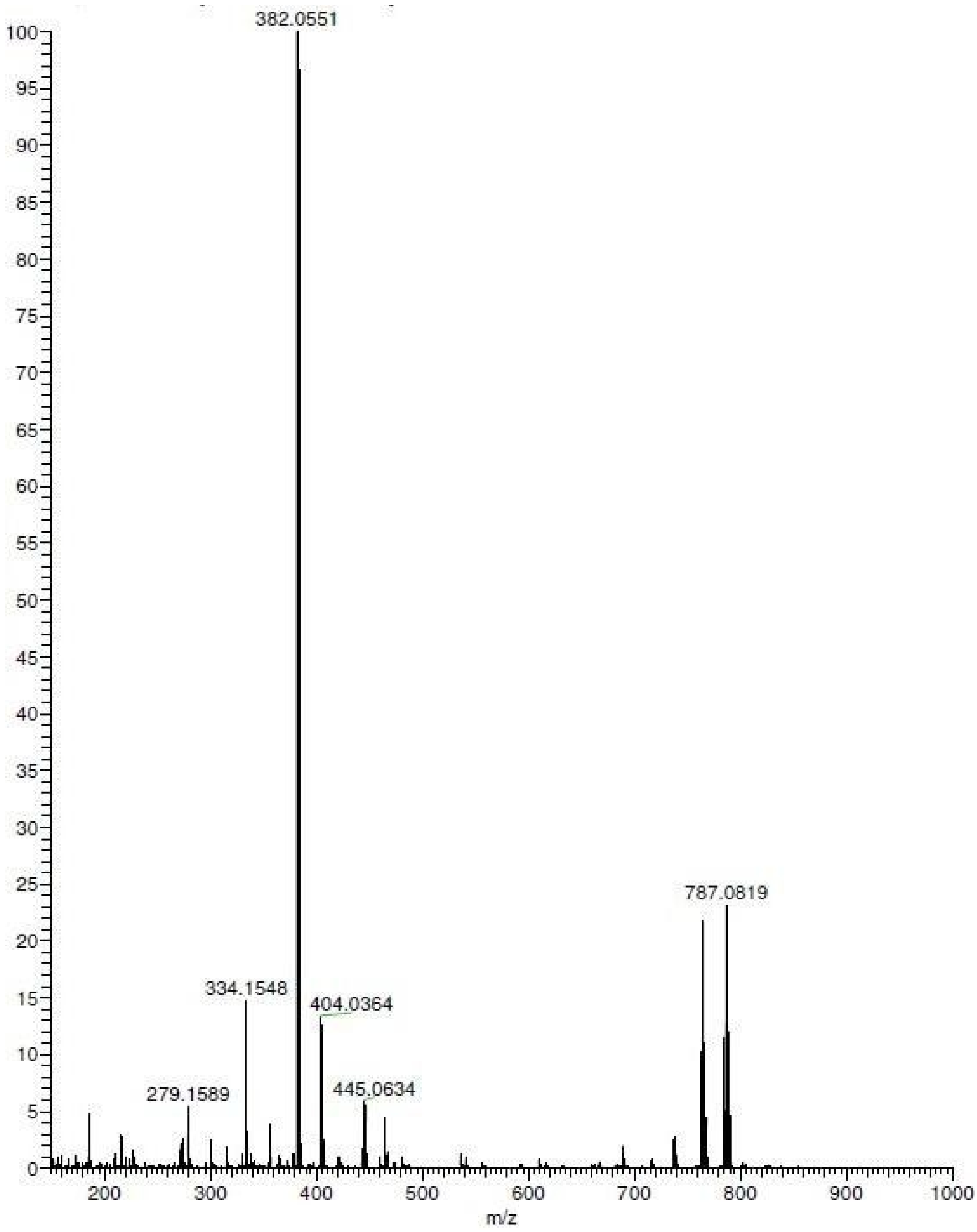

Figure S4. HR-MS spectrum of [Ni(L1)$_2$] (2) in acetonitrile. Calculated: m/z 360.0647 [M-L]$^+$; Found: m/z 382.0551 [M-L-H+Na]$^+$

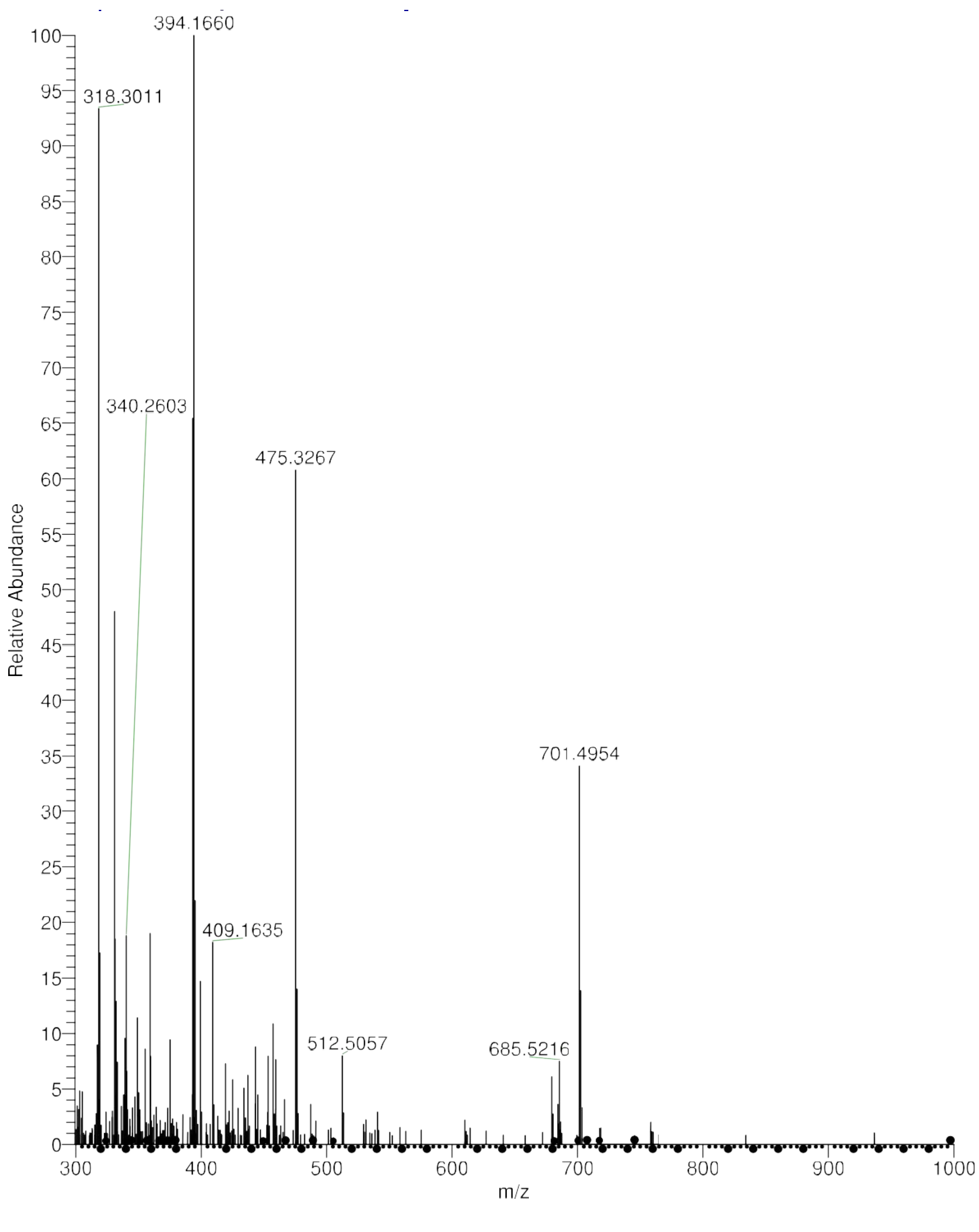

Figure S5. HR-MS spectrum of [Ni(L2)$_2$] (3) in acetonitrile. Calculated: m/z 394.0257 [M-L]$^+$; Found: m/z 394.1660 [M-L]$^+$.

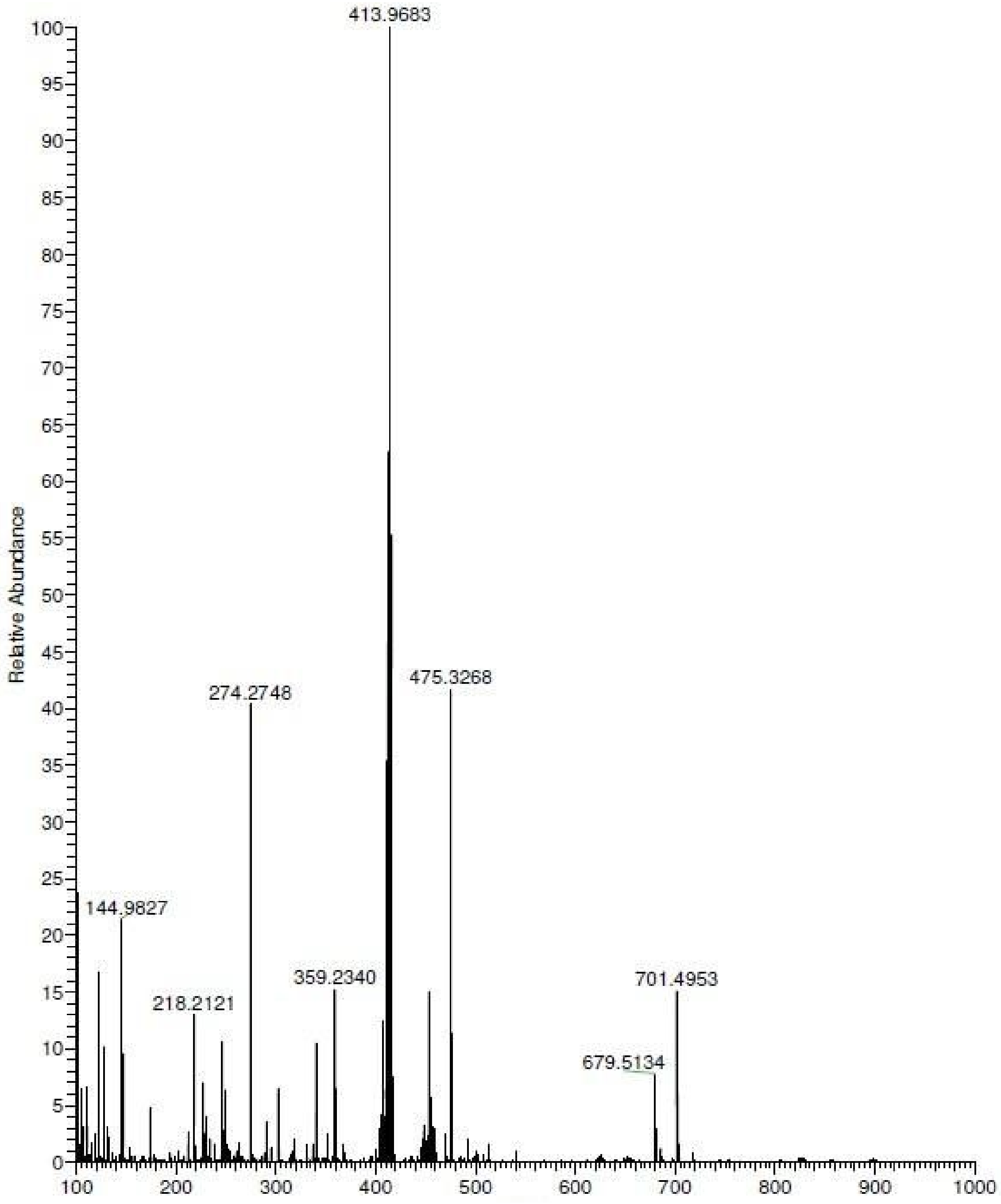

Figure S6. HR-MS spectrum of [Ni(L3)$_2$] (4) in acetonitrile. Calculated: m/z 390.0752 [M-L]$^+$; Found: m/z 413.0650 [M-L+Na]$^+$.

6. Stability studies of the complexes

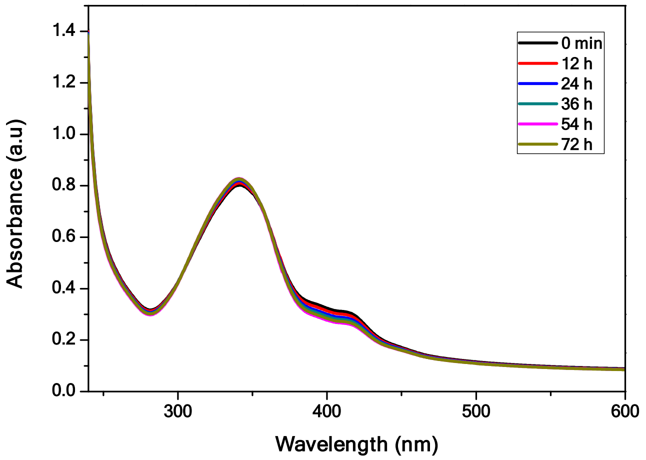

COMPLEX 2

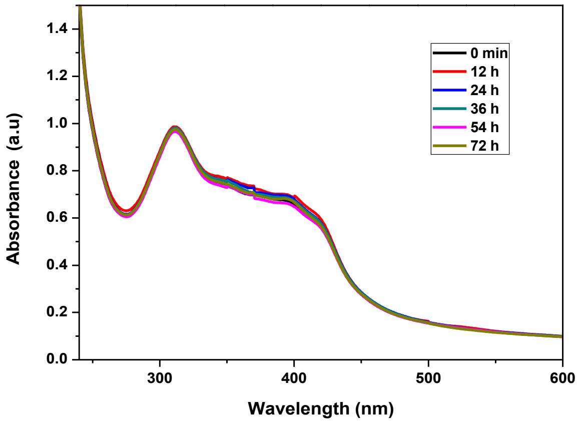

COMPLEX 3

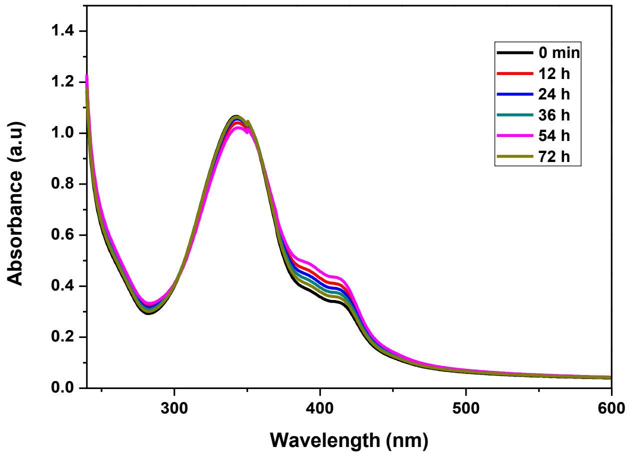

COMPLEX 4

Figure S7. Stability studies of the complexes (2-4) in 1% DMSO in PBS solution at various time intervals (0 - 72 h)

7. REFERANCES

1. A. Kent and D.M. Neil, *J. Chem. Soc.,* **1938**, 8
2. G. M. Sheldrick, *Acta Crystallogr., Sect. A: Found. Crystallogr.***2008**, *64*, 112.
3. L. Farrugia. *J. Appl. Crystallogr*., **1997**, *30*, 565.
4. (a) R. K. Gupta, G. Sharma, R. Pandey, A. Kumar, B. Koch, P. Z. Li, Q. Xu, D. S. Pandey, *Inorg. Chem*., **2013**, *52*, 13984. (b) R. K. Gupta, G. Sharma, R. Pandey, R. Prasad, B. Koch, S. Srikrishna, P. Z. Li, Q. Xu, D. S. Pandey. *Inorg. Chem.,***2013**, *52*, 3687.
